# Long-term mortality prediction after operations for type A ascending aortic dissection

**DOI:** 10.1186/1749-8090-5-42

**Published:** 2010-05-25

**Authors:** Francesco Macrina, Paolo E Puddu, Alfonso Sciangula, Marco Totaro, Fausto Trigilia, Mauro Cassese, Michele Toscano

**Affiliations:** 1 Department of the Heart and Great Vessels "Attilio Reale", Complex Operative Unit of Cardiac Surgery, University of Rome "La Sapienza", Rome, Italy; 2 Department of the Heart and Great Vessels "Attilio Reale", Complex Operative Unit of Biotechnologies Applied to Cardiovascular Diseases, University of Rome "La Sapienza", Rome, Italy; 3 Department of Cardiothoracic Surgery and Cardiology, Sant'Anna Hospital, Catanzaro, Italy

## Abstract

**Background:**

There are few long-term mortality prediction studies after acute aortic dissection (AAD) Type A and none were performed using new models such as neural networks (NN) or support vector machines (SVM) which may show a higher discriminatory potency than standard multivariable models.

**Methods:**

We used 32 risk factors identified by Literature search and previously assessed in short-term outcome investigations. Models were trained (50%) and validated (50%) on 2 random samples from a consecutive 235-patient cohort. NN were run only on patients with complete data for all included variables (N = 211); SVM on the overall group. Discrimination was assessed by receiver operating characteristic area under the curve (AUC) and Gini's coefficients along with classification performance.

**Results:**

There were 84 deaths (36%) occurring at 564 ± 48 days (95%CI from 470 to 658 days). Patients with complete variables had a slightly lower death rate (60 of 211, 28%). NN classified 44 of 60 (73%) dead patients and 147 of 151 (97%) long-term survivors using 5 covariates: immediate post-operative chronic renal failure, circulatory arrest time, the type of surgery on ascending aorta plus hemi-arch, extracorporeal circulation time and the presence of Marfan habitus. Global accuracies of training and validation NN were excellent with AUC respectively 0.871 and 0.870 but classification errors were high among patients who died. Training SVM, using a larger number of covariates, showed no false negative or false positive cases among 118 randomly selected patients (error = 0%, AUC 1.0) whereas validation SVM, among 117 patients, provided 5 false negative and 11 false positive cases (error = 22%, AUC 0.821, p < 0.01 versus NN results). An html file was produced to adopt and manipulate the selected parameters for practical predictive purposes.

**Conclusions:**

Both NN and SVM accurately selected a few operative and immediate post-operative factors and the Marfan habitus as long-term mortality predictors in AAD Type A. Although these factors were not new per se, their combination may be used in practice to index death risk post-operatively with good accuracy.

## Background

Type A acute aortic dissection (AAD) requires emergency replacement of the ascending aorta and/or the aortic arch with or without aortic valve replacement and in-hospital mortality ranges from 7 to 30% in recent series [1,2]. Among 526 patients enrolled from 1996 to 2001 by the International Registry of AAD investigators, 30-day mortality was 25.1% on average [1]. A large list of pre-, intra- and immediate post-operative factors may independently contribute to increase the mortality risk at short-term (see [2] for extensive review). These include: history of aortic valve replacement, migrating chest pain, hypotension and/or shock, cardiac tamponade, limb ischemia, the length of extracorporeal circulation and chronic renal failure. There has also been an effort to investigate whether surgical techniques may contribute to modify the risk; however inconsistent results were obtained as to the role of retrograde, anterograde or selective cerebral perfusion after circulatory arrest [1,2]. More recently, anatomo-surgical parameters [3] and biological indexes, such as D-dimer values above a given threshold [4], were assessed as diagnostic tools, but no study was performed to clarify their potential predictive role. On the other hand, it is largely unknown whether the assessed short-term risk factors may also predict long-term (say 1- to 2-year) mortality in Type A AAD patients.

Aim of the study was therefore to see whether selected risk factors assessed previously for prediction of 30-day mortality risk among Type A AAD patients [1,2], may also contribute to index long-term prediction using neural networks known to have a larger global accuracy as compared to standard models such as logistic regression [2,5]. In addition, to improve discrimination between cases and non cases [6], which is essential once new risk equations are tested in general and in cardiac surgical outcome studies [7-10] in particular, support vector machines (SVM) were also used [11,12] for the first time on this material.

## Methods

### Cohort and Risk Factors

There were 235 consecutive patients undergoing surgical repair of AAD Type A between January 2002 and late 2008 at the University of Rome "La Sapienza"(n = 143, 61%) and Catanzaro Sant'Anna Hospital (n = 92, 39%), Cardiac Surgical Departments. Diagnosis was made in emergency with computer tomographic (CT) scan and/or trans-esophageal echocardiography. Anesthesia was induced by propofol (1-1.8 γml) and sufentanil (0.35-1 γkg) and maintained by propofol 1-1.8 γml/hr and sufentanil 0.35-0.51 γkg/hr.

For each patient there were 32 potential predictors including demographic characteristics and pre-, operative and immediate post-operative variables including dummies (see Additional File 1 for the definition of mathematical, computational or statistical technicalities) constructed in order to index operative techniques and related complications. These were selected based on a Literature review of studies performed to assess the role of relatively short-term potential predictors [2]. Thus, year of surgery, hospital localization, age, sex and presence of clinically diagnosed high blood pressure and Marfan habitus were considered. Among AAD onset symptoms we coded shock and whether intubation was present at arrival or neurological deficits were present. Previous cardiac surgery was also coded. Among intra-operative variables there were: cross-clamping and total circulatory arrest times in min after extracorporeal perfusion started along with operative techniques (whether ascending aorta plus arch or hemi-arch or plus aortic valve and whether by Bentall or Cabrol, all as dummies versus ascending aorta alone). We also coded whether cerebral perfusion was anterograde, retrograde or both. Immediate post-operative complications were noted for each patient and included: total bleeding in ml, limb ischemia, by clinical and CT documentation, renal complications, including oligo-anuria and continuous hemodialysis, gastrointestinal complications such as bleeding and ischemia, and other complications requiring medical or surgical treatment and cerebral accidents, neurological deficits and coma, by clinical and CT documentation. For the definition of the analysed variables we followed those reported in previous studies [1,2].

Follow-up was performed by periodic visits and/or telephone contacts. Death certificates and all pertinent records were reviewed: time and causes of death were considered and patients alive were censored. For the purpose of the study we concentrate here on all-cause mortality.

### Statistical Analysis

Data are expressed as means ± SD or SE (when appropriate). The selection of potential predictors was done *a priori *based on previous knowledge [2,5,13]. Linear correlation with the outcome variable and information value (that is the relative importance of each covariate) were considered. Follow-up data were investigated by modelling the presence (coded 1) or absence (coded 0) of post-operative mortality using Tiberius Data Mining ^© ^software (version 6.1.5; see http://www.tiberius.biz) to obtain multilayer perceptron (MLP) neural network solutions. These were from a 3-layer network, including the hidden unit containing 2 neurons (one linear and the second non-linear), with 32 input nodes (corresponding to the 32 potential risk factors selected) and one output unit, modelling the dichotomous risk outcome [2,5]. MLP were trained on a randomly selected sub sample (50% of all patients included), preventing over-fitting [14,15]. Validation was performed on the remaining 50%. Gini's coefficient and graph [16] were produced. Receiver operating characteristic (ROC) areas under the curve (AUC) were compared [17,18] between solutions using MedCalc software (version 9.6.3.0; see http://www.medcalcsoftware.com). To run SVM [11] cSVM (version 3.1.0; see http://www.smartlab.dibe.unige.it) was used with optimal C search on 50% of the overall sample. There are similarities between neural networks and SVM since an SVM with a sigmoid kernel is equivalent to a neural network with a sigmoid activation function and one hidden unit, the difference being only the number of neurons, automatically selected by a SVM [12]. A value of p < 0.05 was considered statistically significant in all cases.

## Results

### Univariate contributors

The univariate contribution of the 32 potential risk factors for AAD Type A is shown in Additional File 2, Table S1 among the 235 patients studied (see Additional File 2). These patients were from 2 Cardiac Surgical Centres, one in central and the other in southern Italy, and were followed-up from 8 months to 7 years post operation. There were 84 deaths (36%): 81 (95%) of these were of cardiac origin, whereas the remaining 4 (5%) presented mixed causes, from accidents to cancer and suicide. Deaths occurred at 564 ± 48 (mean ± SE) days (95%CI from 470 to 658 days). To index the relative discrimination between cases and non cases (variable = Status) provided individually by these factors, the table shows the information value, Gini's coefficient and linear correlation. A good information value (> 0.5) is provided by chronic renal failure, bleeding in the first post-operative 24 hours, extracorporeal circulation and circulatory arrest times, age, and dummies for post-operative neurological coma and immediate post-operative dialysis in continuous. Apart bleeding in the first post-operative 24 hours, the other variables present a high linear correlation and a large Gini's coefficient.

### Multivariable contribution by NN

**Figure 1:**
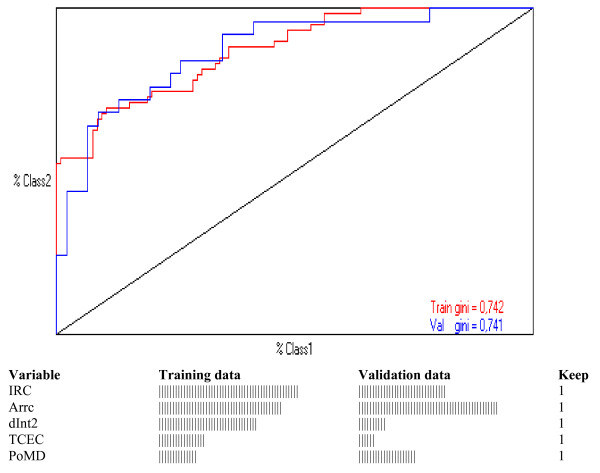
**Receiver operating characteristic plots by randomly selected training (50%) and validation (50%) neural network models on patients with complete variables (N = 211)**. A semi-quantitative graphic presentation of the covariates relevance is presented for training and validation models. Full names of coded variables are reported in Additional File 2, Table S1. Keep = 1 means that covariate may stay in the model. Note that Gini's coefficients are practically identical for training and validation neural network models, respectively 0.742 and 0.741 (ROC AUC: 0.871 and 0.870, respectively). Therefore, training and validation neural network models have a very high, yet similar, accuracy and define a set of 5 predictive covariates useful to index long-term mortality in patients operated for Type A ascending aorta dissection.

There were 211 (90%) patients who had all variables for analysis whereas missing data were seen from 0.4 to 9.4%, depending on the examined variable (Additional File 2, Table S1). There was a slightly lower, not significantly different death rate among patients with complete information for all variables (60 of 211, 28%), than among the overall studied patients. Neural network model classified 44 of 60 (73%) dead patients and 147 of 151 (97%) long-term survivors using 5 covariates: immediate post-operative chronic renal failure, circulatory arrest time, the type of surgery on ascending aorta plus hemi-arch, extracorporeal circulation time and the presence of Marfan habitus. Figure 1 shows a semi-quantitative graphic presentation of these risk factors for training and validation models. The proportions of dead patients identified by neural network were slightly lower in training and validation runs (respectively 69 and 64%) than in the overall study. However, much similar proportions were correctly identified among long-term survivors (respectively 97 and 100%). Of note that global accuracies (as detected by ROC AUC) were extremely high (respectively 0.871 and 0.870).

### Multivariable contribution by SVM

**Figure 2:**
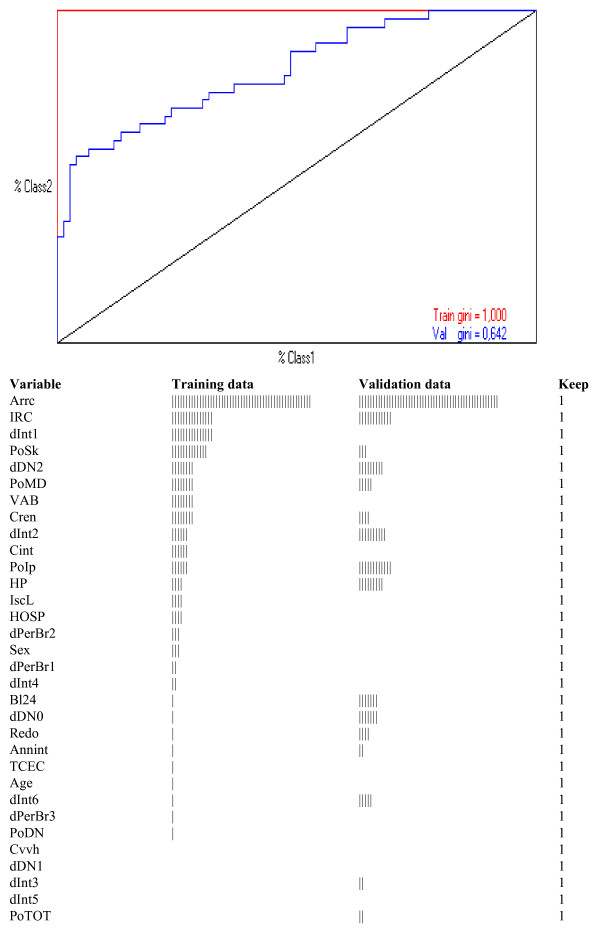
**Receiver operating characteristic plots by randomly selected training (50%) and validation (50%) support vector machine (SVM) with optimal C search on overall study patients (N = 235)**. A semi-quantitative graphic presentation of the covariates relevance is presented for training and validation models. Full names of coded variables are reported in Additional File 2, Table S1. Keep = 1 means that covariate may stay in the model. Using 27 of 32 covariates, Gini's coefficient by training SVM was 1.00 and no false negative or false positive cases were identified among 118 randomly selected AAD Type A patients (error = 0%). However, validation SVM on the remaining 117 patients provided 15 false negative and 11 false positive cases (error = 22%) and the Gini's coefficient was 0.642 (ROC AUC 0.821), which is statistically lower (p < 0.01) than the results obtained by neural network model, shown in Figure 1. Of note that validation and training SVM use different covariates to predict outcome and a relatively different ranked importance. Nevertheless, with both training and validation SVM, apart from extracorporeal circulation time, the other 4 covariates were also selected by neural network models.

Figure 2 shows the results of the SVM run on the overall study group (N = 235), since by this method there is no limitation to confine the analysis to patients with complete data for all variables, as with neural networks. A somewhat different picture is provided by SVM as compared to neural network. First, SVM make use of a larger number of covariates, some of which provided little if any information, yet globally enabled to obtain a Gini's coefficient of 1.00 (using 27 of 32 covariates) with no false negative or false positive cases identified among 118 randomly selected AAD Type A patients (error = 0%). Second, when validation SVM were run on the remaining 117 patients, the Gini's coefficient was 0.642 (with an ROC AUC = 0.821), a statistically lower (p < 0.01) result as compared to those obtained by neural network model. There were 15 false negative and 11 false positive cases (error = 22%) identified. Third, validation and training SVM used different covariates to predict outcome and there was a relatively different ranked importance.

### Variables selected in common by NN and SVM

There were 4 covariates (circulatory arrest time, immediate post-operative chronic renal failure, the type of surgery on ascending aorta plus hemi-arch, and the presence of Marfan habitus) selected in common by neural network models and both training and validation SVM. It is important to consider that a high correlation (r = 0.31) exists between circulatory arrest and extracorporeal circulation times (results not shown).

## Discussion

This is the first investigation to adopt neural networks and support vector machines to assess the relatively long-term predictive role of a quite large series of potential risk factors including pre-operative, operative and immediately post-operative variables in AAD Type A patients. The presence of Marfan habitus, the length of circulatory arrest, an intervention on the ascending aorta plus hemi-arch and immediate post-operative chronic renal failure were the risk factors selected in common by these methods with a very high global accuracy (ROC AUC > 0.82). Although the factors selected were not new, their combination might be used in practice to enable the construction of risk charts whereby levels of risk might be defined. However, it is clear that the corresponding cells of these charts need to contain a sufficient number of cases and non cases, which is presumably possible only after large multi-centre and/or multinational cooperative efforts will be undertaken. The evidence presented here might contribute to stimulate cooperation to reach this aim.

The presented rules provided very good predictive and discrimination properties, however only Marfan habitus was a parameter that could be used pre-operatively. Determination *a priori *about which patients are not candidates for surgery is therefore not possible using the evidence of this investigation. Nevertheless, as there were 2 operative parameters contributing to increase long-term mortality risk, it is important that attention is paid to keep the length of circulatory arrest at the minimal level and to consider that an intervention on the aorta plus hemi-arch conveys an independent risk of lower survival. On the other hand, all efforts should be done to reduce the incidence of post-operative chronic renal failure.

The incidence of AAD Type A has been estimated at from 5 to 30 per million people per year in the United States, which is 880 to 147 times less than the incidence of acute myocardial infarction, but still provides an important clinical problem and sometimes a dilemma for the differentiating difficulties between these presentations [1-3]. Although biological thresholds of plasma molecules such as D-dimer are actively looked for in order to improve diagnosis [4], this may not have an impact on prediction before the results of larger studies are obtained. Therefore, risk profiling remains crucial. Based on results obtained by the IRAD investigators, short-term mortality could be reduced from as high as 58% in medically treated patients to the current average figure of 25.1% (and sometimes less) when surgery is performed [1]. Risk factors may contribute to better management and a more defined risk assessment [1,2]: in-hospital mortality was as high as 31.4% in unstable patients presenting with cardiac tamponade, shock, congestive heart failure, cerebro-vascular accident, stroke, coma, acute myocardial and/or mesenteric ischemia and acute renal failure at the time of operation, whereas stable patients may present with a mortality as low as 16.7%.

In a previous report we investigated 30-day mortality among 208 patients from 2 Italian Centres [2] using a series of demographic, pre-operative, operative and post-operative characteristics, selected from 37 such variables considered in the Literature as potential predictors of short-term mortality after AAD Type A. When logistic or neural network models were produced in one Centre and applied to the data from the second Centre, for external validation [13-15], there were predictors which were selected in common: the presence of pre-operative shock, intubation and neurological symptoms, immediate post-operative presence of dialysis in continuous and the quantity of bleeding in the first 24 hours post-operation. By neural network model only, the length of extracorporeal circulation and post-operative chronic renal failure were detected as independent predictors of 30-day mortality. Different from the IRAD Registry investigators [1] we showed [2] that operative and immediate post-operative factors should be considered to predict short-term mortality. They contributed significantly to obtain a large overall accuracy, which might be explained in part by these factors being continuous [19]. On the other hand, similar to studies investigating predictive performance of short-term mortality after coronary artery bypass surgery [9,10], neural networks had a better performance when compared to standard methods such as logistic regression [2,5].

When the performance and/or reliability of predictive models is limited, or of low sensitivity and specificity, their capability may be hampered to identify high risk subjects who deserve individualized treatment [13]. The neural network method stems [14,15] from its potential for improved predictive performance by exploring, hidden layers to find nonlinearities, interactions and nonlinear interactions among predictors. The attraction of neural networks is quite evident from the impressive growth of results published [15]. However, there are relatively few comparative reports on the performance and accuracy of neural networks, which was assessed only versus multiple logistic function, to predict events in clinical [9] or epidemiological [5,18] cardiovascular studies.

There has been some controversy as to whether new risk predictors, or series of old and newer ones, can add to the prediction of events, including mortality, in terms of clinical utility, impact or discrimination [6]. Although in clinical and epidemiological experiences discrimination metrics (such as ROC AUC) are quite well established methods [2,5,18,20,21], it has been pointed out that ROC AUC are insensitive in comparing models [6], which may be circumvented however by making comparisons with fixed number of covariates [5]. To evaluate and compare predictive risk models there have been therefore new methods to be proposed, based primarily on stratification into clinical categories on the basis of risk and attempts to assess the ability of new models to more accurately reclassify individuals into higher or lower risk strata [22,23]. Risk reclassification for single factors can be then examined by using models with and without each risk factor in turn or measuring the net reclassification improvement, that is the difference in proportions moving up and down risk strata among case patients versus control participants [6,23]. Whatever reclassification method is selected it is important to understand that when length of follow-up differs (as in the present series) among individuals and/or the cohort is relatively small it may be impossible to apply them [6]. Moreover and more importantly, reclassification methods depend on the particular categories used [6]: in our case it is far from established if a 5%, 10%, 20%, 30% or more are adequate categories of long-term risk of AAD Type A. To compare with established experiences in preventive cardiology [20,24] or coronary by-pass surgery [25], the sensitivity and specificity of the abovementioned thresholds should be accurately assessed, which again calls for large amount of data being collected and therefore improved multi-centre collaboration.

## Conclusions

The classification provided by neural network models and related SVM may represent a compromise to cope with the necessity to assess the clinical relevance of variables used for predictive purposes in AAD Type A patients, but also in different areas of research. These methods may also go beyond the classical contention of standard predictive models, namely that only predictors that are statistically significant are typically used [6]. Indeed, with SVM a high discrimination is obtained by using a large number of variables, most with little informative content if used alone. As we have shown, however, it is extremely important not only to train but to validate these methods, which demands further study and the accumulation of very large data sets. Our results may well stimulate these efforts.

An important take-home message for clinicians should be that with neural networks and SVM, by concentrating on a few risk factors such as those described here, it is possible to predict long-term mortality in AAD Type A patients with a global good accuracy. We produced an html tool (see Additional File 3) based on the neural network solution reported here, whereby it is easy to appreciate that increasing from 60 to 80 min the circulatory arrest time, the patient long-term risk category evolves from false (survival) to true (dead) at an assessment strength (roughly the degree of certitude) of 1/3. By further increasing circulatory arrest times to 120 and 180 min, the assessment strengths become 2/3 and almost 1, respectively. Although Surgeons know well and from decades that this is a hardly steerable variable in the clinical practice, a dimensional outcome predictive assessment might be obtained using our tool immediately after the operation is finished, which may have an impact for further clinical decision making. The other variables described in the present study might also be used for predictive assessments so that a very large combination of clinical presentations could be easily modeled.

## Competing interests

The authors declare that they have no competing interests.

## Authors' contributions

FM and PEP participated equally in the design of the study. PEP performed the statistical analysis, drafted the manuscript and coordinated the implementation of it. AS collected the data, obtained the follow-up information and participated in the draft of the manuscript. MT1 participated in the data collection and the draft of the manuscript. FT collected the data, obtained the follow-up information and participated in the statistical analysis and the draft of the manuscript. MC and MT2 conceived of the study, and participated in its design and coordination and helped to draft the manuscript. All authors read and approved the final manuscript.

## Supplementary Material

Additional file 1 Appendix to explain plainly statistical technicalities. Click here for file

Additional file 2 Table S1: Description and univariate contribution of 32 potential risk factors. Click here for file

Additional file 3 **Model2Chronic.html**. This is a tool to use the multivariable assessment of long-term mortality in Type A AAD patient, as obtained in this investigation. Click here for file

## References

[B1] Trimarchi S Nienaber CA Rampoldi V Myrmel T Suzuki T Mehta RH Bossone E Cooper JV Smith DE Menicanti L Frigiola A Oh JK Deeb MG Isselbacher EM Eagle KA International Registry of Acute Aortic Dissection Investigators Contemporary results of surgery in acute type A aortic dissection: The International registry of Acute Aortic Dissection experience J Thorac Cardiovasc Surg 2005 129 112 122 10.1016/j.jtcvs.2004.09.005 15632832

[B2] Macrina F Puddu PE Sciangula A Trigilia F Totaro M Miraldi F Toscano F Cassese M Toscano M Artificial neural networks versus multiple logistic regression to predict 30-day mortality after operations for Type A ascending aortic dissection Open Cardiovasc Med J 2009 3 81 95 10.2174/1874192400903010081 19657459 PMC2720513

[B3] Homme JL Aubry MC Edwards WD Bagniewski SM Shane Pankratz V Kral CA Tazelaar HD Surgical pathology of the ascending aorta: a clinicopathologic study of 513 cases Am J Surg Pathol 2006 30 1159 1168 16931961 10.1097/01.pas.0000213270.38091.69

[B4] Suzuki T Distante A Zizza A Trimarchi S Villani M Salerno Uriarte JA De Luca Tapputi Schinosa L Renzulli A Sabino A Nowak R Birkhahn R Hollande JE Counselman F Vijayendran R Bossone E Eagle K for the IRAD-Bio Investigators Diagnosis of acute aortic dissection by D-dimer: the international registry of acute aortic dissection substudy on biomarkers (IRAD-Bio) experience Circulation 2009 119 2702 2707 10.1161/CIRCULATIONAHA.108.833004 19433758

[B5] Puddu PE Menotti A Artificial neural network versus multiple logistic function to predict 25-year coronary heart disease mortality in the Seven Countries Study Eur J Cardiovasc Prev Rehabil 2009 16 583 591 10.1097/HJR.0b013e32832d49e1 19602982

[B6] Cook NR Ridker PM Advances in measuring the effect of individual predictors of cardiovascular risk: the role of reclassification measures Ann Intern Med 2009 150 795 802 19487714 10.7326/0003-4819-150-11-200906020-00007 PMC2782591

[B7] Orr RK Use of a probabilistic neural network to estimate the risk of mortality after surgery Med Decis Making 1997 17 178 185 10.1177/0272989X9701700208 9107613

[B8] Shahian DM Blackstone EH Edwards FH Grover FL Grunkemeier GL Naftel DC Nashef SAM Nugent WC Peterson ED Cardiac surgery risk models: a position article Ann Thorac Surg 2004 78 1868 1877 10.1016/j.athoracsur.2004.05.054 15511504

[B9] Nilsson J Ohlsson M Thulin L Höglund P Nashef SAM Brandt J Risk factor identification and mortality prediction in cardiac surgery using artificial neural networks J Thorac Cardiovasc Surg 2006 132 12 19 10.1016/j.jtcvs.2005.12.055 16798296

[B10] Goto M Kohsaka S Aoki N Lee VV Elayda MA Wilson JM Risk stratification after successful coronary revascularization Cardiovasc Revasc Med 2008 9 132 139 10.1016/j.carrev.2008.03.005 18606375

[B11] Cristianini N Shawe-Taylor J An introduction to support vector machines and other kernel-based learning methods 2000 Cambridge, Cambridge University Press

[B12] Shawe-Taylor J Cristianini N Kernel methods for pattern analysis 2004 Cambridge, Cambridge University Press

[B13] May M Commentary: improved coronary risk prediction using neural networks Int J Epidemiol 2002 31 1262 1263 10.1093/ije/31.6.1262

[B14] Tu JV Advantages and disadvantages of using artificial neural networks versus logistic regression for predicting medical outcomes J Clin Epidemiol 1996 49 1225 1231 10.1016/S0895-4356(96)00002-9 8892489

[B15] Dayhoff JE DeLeo JM Artificial neural networks. Opening the black box Cancer 2001 91 1615 1635 10.1002/1097-0142(20010415)91:8+<1615::AID-CNCR1175>3.0.CO;2-L 11309760

[B16] Gini C Measurement of inequality of incomes The Economic Journal 1921 31 124 126 10.2307/2223319

[B17] Hanley JA McNeil BJ A method of comparing the areas under receiver operating characteristic curves derived from the same cases Radiology 1983 148 839 843 6878708 10.1148/radiology.148.3.6878708

[B18] Voss R Cullen P Schulte H Assmann G Prediction of risk of coronary events in middle-aged men in the prospective cardiovascular Münster study (PROCAM) using neural networks Int J Epidemiol 2002 31 1253 1262 10.1093/ije/31.6.1253 12540731

[B19] Altman DG Categorizing continuous variables Br J Cancer 1991 64 975 1931629 10.1038/bjc.1991.441 PMC1977443

[B20] Conroy RM Pyörälä K Fitzgerald AP Sans S Menotti A De Backer G De Bacquer D Ducimetière P Jousilahti P Keil U Njølstad I Oganov RG Thomsen T Tunstall-Pedoe H Tverdal A Wedel H Whincup P Wilhelmsen L Graham IM on behalf of the SCORE project group Estimation of ten-year risk of fatal cardiovascular disease in Europe: the SCORE project Eur Heart J 2003 24 987 1003 10.1016/S0195-668X(03)00114-3 12788299

[B21] Sciangula A Puddu PE Schiariti M Acconcia MC Missiroli B Papalia U Gaudio C Martinelli G Cassese M Comparative application of multivariate models developed in Italy and Europe to predict early (28 days) and late (1 year) postoperative death after on- or off-pump coronary artery bypass grafting Heart Surg Forum 2007 10 E258 E266 10.1532/HSF98.20071021 17599870

[B22] Cook NR Use and misuse of the receiver operating characteristic curve in risk prediction Circulation 2007 115 928 935 10.1161/CIRCULATIONAHA.106.672402 17309939

[B23] Pencina MJ D'Agostino RB Sr D'Agostino RB Jr Vasan RS Evaluating the added predictive ability of a new marker: from area under the ROC curve to reclassification and beyond Stat Med 2008 27 157 172 10.1002/sim.2929 17569110

[B24] Menotti A Puddu PE Lanti M Comparison of the Framingham risk function based coronary risk with risk function from an Italian population study Eur Heart J 2000 21 365 370 10.1053/euhj.1999.1864 10666350

[B25] Puddu PE Brancaccio G Leacche M Monti F Lanti M Menotti A Gaudio C Papalia U Marino B on behalf of the OP-RISK Study Group Prediction of early and delayed postoperative deaths after coronary artery bypass surgery in Italy. Multivariate prediction based on Cox and logistic models and a chart based on the accelerated failure time model Ital Heart J 2002 3 166 181 11974661

