# Supplementary material for: Long-term mortality prediction after operations for type A ascending aortic dissection
Source: J Cardiothorac Surg. 2010 May 25;5:42. doi: 10.1186/1749-8090-5-42 (PMC2902218; doi:10.1186/1749-8090-5-42)
Supplement: Additional file 1 — Appendix to explain plainly statistical technicalities. [file 1749-8090-5-42-S1.DOC]

**Appendix**

The relatively complex terminology adopted in the domain of artificial neural networks prompted some lay translations for the non-initiated to fully understand the approximate correspondence with more familiar terms used for multiple logistic function [14]. Here is a list of comparative terms between these approaches:

| **Standard Multivariable Methods** | **Neural Networks** | |
| --- | --- | --- |
| Independent (predictor) variable | | Input |
| Dependent (outcome) variable | | Output |
| Predicted probability | | Transformed probability |
| Regression coefficient | | Weight in the connection |
| Intercept parameter | | Bias weight |
| Parameter estimation | | Learning |
| Observation | | Training case, pattern |

Interested readers are also referred to the important review article by Dayhoff and De Leo [15] for extensive applications of neural network models to various medical disciplines, along with a more recent paper where we addressed specifically applications to cardiology [5]. Here is a concise translation in plane terms of the significance of specialized terminology in the area of neural network modelling and support vector machines and related mathematical and computational areas:

**Neural networks:** are mathematical or computational models that try to simulate the structure and/or functional aspects of biological networks. An interconnected group of artificial neurons processes information using connectionist-driven computations. They are mostly adaptive systems that change their structure based on external or internal information that flows through the network during the learning phase. In more general terms they are non-linear statistical tools for data analysis and they find patterns in data by modeling complex relationships between inputs and outputs. The core element stay in a network of simple processing elements (neurons), exhibiting complex global behavior: they determine the connections between the processing elements and element parameters. In a neural network model, simple nodes (called "neurodes", or "processing elements") are connected together to form a network of nodes. While a neural network does not have to be adaptive per se, its practical use comes with algorithms designed to alter the strength (weights) of the connections in the network to produce a desired signal flow. Non-linear relationship are also medelled at the level of hidden layers whose number could be varied, although it is generally kept under two.

**Support vector machines**: are a set of related supervised learning methods used for classification and regression. Given a set of training data belonging to two distinct subsets, this method automatically assigns further data to one of the subsets. A support vector machine constructs a hyper-plane or set of hyper-planes in a high or infinite dimensional space, which can be used for classification, regression or other tasks. Intuitively, a good separation is achieved by the hyper-plane that has the largest distance to the nearest training data points of any class (so-called functional margin), since in general the larger the margin the lower the generalization error of the classifier. Suppose some given data points each belong to one of two classes, and the goal is to decide which class a new data point will be in. In the case of support vector machines, a data point is viewed as a *p*-dimensional vector (a list of *p* numbers), and we want to know whether we can separate such points with a *p* − 1-dimensional hyper-plane (linear classifier). There are many hyper-planes that might classify the data. One reasonable choice as the best hyper-plane is the one that represents the largest separation, or margin, between the two classes. So we choose the hyper-plane so that the distance from it to the nearest data point on each side is maximized. If such a hyperplane exists, it is known as the maximum margin hyper-plane.

**Hyper-plane:** is a concept in geometry. It is a generalization of the concept of a plane into a different number of dimensions. Analogous with a plane which defines a two-dimensional subspace in a three-dimensional space, a hyper-plane defines a *k*-dimensional subspace within an *n*-dimensional space, where *k*<*n*. A line, for example, is a one-dimensional hyper-plane in a space with any number of dimensions. High dimensional hyper-planes are difficult to visualize, but they share many mathematical properties in common with regular lines and planes.

**Classification error in Statistics:** statistical classification is a supervised machine learning procedure in which individual items are placed into groups based on quantitative information on one or more characteristics inherent in the items (referred to as traits, variables, characters, etc) and based on a training set of previously labeled items. Classifier performance depends greatly on the characteristics of the data to be classified. There is no single classifier that works best on all given problems. Various empirical tests have been performed to compare classifier performance and to find the characteristics of data that determine classifier performance. Determining a suitable classifier for a given problem is however still more an art than a science. The measures precision and recall are popular metrics used to evaluate the quality of a classification system. ROC curves (see later) have also been used to evaluate the tradeoff between true- and false-positive rates of classification algorithms. The most widely used classifiers are the neural networks (multi-layer perceptron), support vector machines, Gaussian mixture model, Gaussian, naïve Bayes, and decision trees.

**Function:** is a relation between a given set of elements (the domain) and another set of elements (the co-domain), which associates each element in the domain with exactly one element in the co-domain. The elements so related can be any kind of thing (words, objects, qualities) but are typically mathematical quantities such as real numbers. There are many ways to represent or visualize functions: a function may be described by a formula, by a plot or graph, by an algorithm that computes it, by arrows between objects, or by a description of its properties. In applied disciplines, functions are frequently specified by tables of values or by formulae. In a setting where outputs of functions are numbers, functions may be added and multiplied, yielding new functions. An important operation on functions, which distinguishes them from numbers, is composition of functions. The composite function is obtained by using the output of one function as the input of another. This operation provides the theory of functions with its most powerful structure.

**Multilayer perceptron:** is a feedforward artificial neural network model that maps sets of input data onto a set of appropriate output. It is a modification of the standard linear perceptron in that it uses three or more layers of neurons (nodes) with nonlinear activation functions, and is more powerful than the perceptron in that it can distinguish data that is not linearly separable, or separable by a hyper-plane. If a multilayer perceptron consists of a linear activation function in all neurons, that is, a simple on-off mechanism to determine whether or not a neuron fires, then it is easily proved with linear algebra that any number of layers can be reduced to the standard two-layer input-output model. What makes a multilayer perceptron different is that each neuron uses a non-linear activation function which was developed to model the frequency of action potentials, or firing, of biological neurons in the brain. This function is modeled in several ways, but must always be normalizable and differentiable. The two main activation functions used in current applications are both sigmoids in which the former function is a hyperbolic tangent which ranges from -1 to 1, and the latter is equivalent in shape but ranges from 0 to 1. Here *y_i_* is the output of the *i*th node (neuron) and *v_i_* is the weighted sum of the input synapses. More specialized activation functions include radial basis functions. The multilayer perceptron consists of an input and an output layer with one or more hidden layers of nonlinearly-activating nodes. Each node in one layer connects with a certain weight *w_ij_* to every node in the following layer. Learning occurs in the perceptron by changing connection weights after each piece of data is processed, based on the amount of error in the output compared to the expected result. This is an example of supervised learning, and is carried out through backpropagation, a generalization of the least mean squares algorithm in the linear perceptron.

**Sigmoid activation:** many natural processes and complex system learning curves display a history dependent progression from small beginnings that accelerates and approaches a climax over time. A sigmoid curve is produced by a mathematical function having an "S" shape. Often, sigmoid function refers to the special case of the logistic function. In its simplest form, this function is binary (that is: either the neuron is firing or not). The function looks like φ(*v_i_*) = *U*(*v_i_*), where *U* is the Heaviside step function. A line of positive slope may also be used to reflect the increase in firing rate that occurs as input current increases. The function would then be of the form φ(*v_i_*) = μ*v_i_*, where μ is the slope. This activation function is linear, and therefore has the same problems as the binary function. In addition, networks constructed using this model have unstable convergence because neuron inputs along favored paths tend to increase without bound, as this function is not normalizable. All problems mentioned above can be handled by using a normalizable sigmoid activation function. One realistic model stays at zero until input current is received, at which point the firing frequency increases quickly at first, but gradually approaches an asymptote at 100% firing rate. Mathematically, this looks like φ(*v_i_*) = *U*(*v_i_*)tanh(*v_i_*), where the hyperbolic tangent function can also be any sigmoid. This behavior is realistically reflected in the neuron, as neurons cannot physically fire faster than a certain rate. This model runs into problems, however, in computational networks as it is not differentiable, a requirement in order to calculate backpropagation. The final model that is used in multilayer perceptrons is a sigmoidal activation function in the form of a hyperbolic tangent. Two forms of this function are commonly used: φ(*v_i_*) = tanh(*v_i_*) whose range is normalized from -1 to 1, and φ(*v_i_*) = (1 + exp( − *v_i_*)) ^− 1^ is vertically translated to normalize from 0 to 1. The latter model is often considered more biologically realistic.

**Training and Validation:** in machine learning, early stopping is a form of regularization used when a machine learning model (such as a neural network) is trained by on-line gradient descent. In early stopping, the training set is split into a new training set and a validation set. This is the procedure we adopted in the present study where the total set was randomly split in 50% training and 50% validation sets. Gradient descent is applied to the new training set. After each sweep through the new training set, the network is evaluated on the validation set. When the performance with the validation test stops improving, the algorithm halts. The network with the best performance on the validation set is then used for actual testing, with a separate set of data (the validation set is used in learning to decide when to stop). This technique is a simple but efficient hack to deal with the problem of overfitting.

**Overfitting**: is a phenomenon in which a learning system, such as a neural network gets very good at dealing with one data set at the expense of becoming very bad at dealing with other data sets. Early stopping is a very common practice in neural network training and often produces networks that generalize well. However, while often improving the generalization it does not do so in a mathematically well-defined way. Overfitting occurs when a statistical model describes random error or noise instead of the underlying relationship. It generally occurs when a model is excessively complex, such as having too many degrees of freedom, in relation to the amount of data available. A model which has been overfit will generally have poor predictive performance, as it can exaggerate minor fluctuations in the data. The potential for overfitting depends not only on the number of parameters and data but also the conformability of the model structure with the data shape, and the magnitude of model error compared to the expected level of noise or error in the data. Even when the fitted model does not have unusually many degrees of freedom, it is to be expected that the fitted relationship will appear to perform less well on a new data set than on the data set used for fitting. In order to avoid overfitting, it is necessary to use additional techniques (e.g. cross-validation, regularization, early stopping, Bayesian priors or model comparisons). Early stopping and cross-validation were selected here.

**Cross-validation:** sometimes called rotation estimation is a technique for assessing how the results of a statistical analysis will generalize to an independent data set. It is mainly used in settings where the goal is prediction, and one wants to estimate how accurately a predictive model will perform in practice. One round of cross-validation involves partitioning a sample of data into complementary subsets, performing the analysis on one subset (called the training set), and validating the analysis on the other subset (called the validation set or testing set). To reduce variability, multiple rounds of cross-validation could be performed using different partitions, and the validation results are averaged over the rounds (see later in: Bootstrap Technique). Cross-validation is important in guarding against testing hypotheses suggested by the data (also referred to as Type III errors), especially where further samples are hazardous, costly or impossible to collect. Cross-validation is therefore a way to predict the fit of a model to a hypothetical validation set when an explicit validation set is not available. As the present study was undertaken by mixing-up the data from 2 Centres whose individual numerical extent was scant, it was decided to adopt cross-validation rather than fitting the model in one Centre and validating it in the second one (i.e. external validation). When the external validation method was selected in case of short-term predictors of TypeA AAD [2], the training neural network model derived in the first Centre performed very well in the second Centre.

**Receiver Operating Characteristic (ROC) Curves and their comparison:** In signal detection theory, a ROC curve, is a graphical plot of the sensitivity versus (1 − specificity) for a binary classifier system as its discrimination threshold is varied. The ROC can also be represented equivalently by plotting the fraction of true positives versus the fraction of false positives. Also known as a Relative Operating Characteristic curve, because it is a comparison of two operating characteristics (i.e. the rates of true positives and false positives) as the criterion changes. ROC analysis provides tools to select possibly optimal models and to discard suboptimal ones independently from (and prior to specifying) the cost context or the class distribution. ROC analysis is related in a direct and natural way to cost/benefit analysis of diagnostic decision making. The ROC curve was first used during [World War II](http://en.wikipedia.org/wiki/World_War_II) for the analysis of [radar signals](http://en.wikipedia.org/wiki/Radar) before it was employed in [signal detection theory](http://en.wikipedia.org/wiki/Signal_detection_theory). Following the [attack on Pearl Harbor](http://en.wikipedia.org/wiki/Attack_on_Pearl_Harbor) in 1941, the United States army began new research to increase the prediction of correctly detected Japanese aircraft from their radar signals. In the 1950s, ROC curves were employed in [psychophysics](http://en.wikipedia.org/wiki/Psychophysics) to assess human (and occasionally non-human animal) detection of weak signals. In [medicine](http://en.wikipedia.org/wiki/Medicine), ROC analysis has been extensively used in the evaluation of [diagnostic tests](http://en.wikipedia.org/wiki/Diagnostic_test). ROC curves are also used extensively in [epidemiology](http://en.wikipedia.org/wiki/Epidemiology) and [medical research](http://en.wikipedia.org/wiki/Medical_research) and are frequently mentioned in conjunction with [evidence-based medicine](http://en.wikipedia.org/wiki/Evidence-based_medicine). In radiology, ROC analysis is a common technique to evaluate new radiology techniques. In the social sciences, ROC analysis is often called the ROC Accuracy Ratio, a common technique for judging the accuracy of default probability models. ROC curves also proved useful for the evaluation of [machine learning](http://en.wikipedia.org/wiki/Machine_learning) techniques. The first application of ROC in machine learning was by Spackman who demonstrated the value of ROC curves in comparing and evaluating different classification [algorithms](http://en.wikipedia.org/wiki/Algorithm). Several tests have been proposed to compare ROC curves and softwares exist enabling these tests to be performed easily.

**ROC AUC and Gini plots:** these are exactly the same. It is just a different fraction of the curve. Gini is the area between the curve and the diagonal. AUC is the total area under the curve. Thus, AUC = (0.5 * gini) + 0.5. We used Gini since this is the metric selected by Tiberius software. Corrado Gini was an Italian [statistician](http://en.wikipedia.org/wiki/Statistician), [demographer](http://en.wikipedia.org/wiki/Demography) and [sociologist](http://en.wikipedia.org/wiki/Sociology) who developed the [Gini coefficient](http://en.wikipedia.org/wiki/Gini_coefficient), a measure of the [income](http://en.wikipedia.org/wiki/Income) inequality in a [society](http://en.wikipedia.org/wiki/Society). He was born on 1884 in [Motta di Livenza](http://en.wikipedia.org/wiki/Motta_di_Livenza), near [Treviso](http://en.wikipedia.org/wiki/Treviso) and died in Rome 1965. He became a professor at the University of Rome in 1925. At the University, he founded a lecture course on sociology, which he maintained until his retirement. The Gini coefficient is a [measure of statistical dispersion](http://en.wikipedia.org/wiki/Statistical_dispersion#Measures_of_statistical_dispersion) most prominently used as a [measure of inequality of income distribution](http://en.wikipedia.org/wiki/Income_inequality_metrics) or [inequality of wealth distribution](http://en.wikipedia.org/wiki/Wealth_condensation). It is defined as a [ratio](http://en.wikipedia.org/wiki/Ratio) with values between 0 and 1: A low Gini coefficient indicates more equal income or wealth distribution, while a high Gini coefficient indicates more unequal distribution: 0 corresponds to perfect equality (everyone having exactly the same income) and 1 corresponds to perfect inequality (where one person has all the income, while everyone else has zero income). The Gini coefficient requires that no one have a negative net income or wealth. Worldwide, Gini coefficients range from approximately 0.232 in [Denmark](http://en.wikipedia.org/wiki/Denmark) to 0.707 in [Namibia](http://en.wikipedia.org/wiki/Namibia) although not every country has been assessed. The Gini coefficient was published in his 1912 paper "Variabilità e mutabilità”. The concept is useful in studies of [biodiversity](http://en.wikipedia.org/wiki/Biodiversity), where cumulative proportion of species is plotted against cumulative proportion of individuals. The Gini index is the Gini coefficient expressed as a percentage. Thus Denmark's Gini index is 23.2. On the other hand, the AUC is equal to the probability that a classifier will rank a randomly chosen positive instance higher than a randomly chosen negative one. It can be shown that the area under the ROC curve is equivalent to the [Mann-Whitney U](http://en.wikipedia.org/wiki/Mann-Whitney_U), which tests for the median difference between scores obtained in the two groups considered if the groups are of continuous data. It is also equivalent to the [Wilcoxon test of ranks](http://en.wikipedia.org/wiki/Wilcoxon_signed-rank_test). The AUC has been found to be related to the [Gini coefficient](http://en.wikipedia.org/wiki/Gini_coefficient)(G) by the following formula G_1_ + 1 = 2xAUC. The machine learning community most often uses the ROC AUC statistic for model comparison. This measure can be interpreted as the probability that when we randomly pick one positive and one negative example, the classifier will assign a higher score to the positive example than to the negative. In engineering, the area between the ROC curve and the no-discrimination line is often preferred, because of its useful mathematical properties as a [non-parametric statistic](http://en.wikipedia.org/wiki/Non-parametric_statistic). This area is often simply known as the discrimination. The discrimination area used by engineers is precisely Gini coefficient.

**Transformation:** it refers to the application of a deterministic mathematical function to each point in a data set. Each data point *z_i_* is replaced with the transformed value *y_i_* = *f*(*z_i_*), where *f* is a function. Transforms are usually applied so that the data appear to more closely meet the assumptions of a statistical inference procedure that is to be applied, or to improve the interpretability or appearance of graphs. Nearly always, the function that is used to transform the data is invertible, and generally is continuous. The transformation is usually applied to a collection of comparable measurements. For example, if we are working with data on peoples' incomes in some currency unit, it would be common to transform each person's income value by the logarithm function. It is not always necessary or desirable to transform a data set to resemble a normal distribution. However, if symmetry or normality are desired, they can often be induced through one of the power transformations. To assess whether normality has been achieved, a graphical approach is usually more informative than a formal statistical test. Alternatively, rules of thumb based on the sample skewness and kurtosis have also been proposed, such as having skewness in the range of −0.8 to 0.8 and kurtosis in the range of −3.0 to 3.0.

**Dummy variables:** also known as indicator variables or just dummy, is one that takes the values 0 or 1 to indicate the absence or presence of some categorical effect that may be expected to shift the outcome. For example, in econometric time series analysis, dummy variables may be used to indicate the occurrence of wars, or major strikes. It could thus be thought of as a truth value represented as a numerical value 0 or 1 (as is sometimes done in computer programming). Use of dummy variables usually increases model fit (coefficient of determination), but at a cost of fewer degrees of freedom and loss of generality of the model. Too many dummy variables result in a model that does not provide any general conclusions. Dummy variables may be extended to more complex cases. When there are dummies in all observations, the constant term has to be excluded. If a constant term is included in the regression, it is important to exclude one of the dummy variables from the regression, making this the base category against which the others are assessed. If all the dummy variables are included, their sum is equal to 1 (which stands for the variable *X*_0_ to the constant term *B*_0_), resulting in perfect multicolinearity which is referred to as the dummy variable trap. In this study, the dummy variable trap was specifically prevented.

**Bootstrap Technique:** It was Bradley Efron (born 1938) to introduce in 1979 the bootstrap re-sampling technique, which has had a major impact in the field of statistics and virtually every area of statistical application. The bootstrap was one of the first computer-intensive statistical techniques, replacing traditional algebraic derivations with data-based computer simulations. Bootstrapping is the practice of estimating properties of an estimator (such as its variance) by measuring those properties when sampling from an approximating distribution. One standard choice for an approximating distribution is the empirical distribution of the observed data. In the case where a set of observations can be assumed to be from an independent and identically distributed population, this can be implemented by constructing a number of re-samples of the observed dataset (and of equal size to the observed dataset), each of which is obtained by random sampling with replacement from the original dataset. The advantage of bootstrapping over analytical methods is its great simplicity. It is straightforward to apply the bootstrap to derive estimates of standard errors and confidence intervals for complex estimators of complex parameters of the distribution, such as percentile points, proportions, odds ratio, and correlation coefficients. The disadvantage of bootstrapping is that while (under some conditions) it is asymptotically consistent, it does not provide general finite-sample guarantees, and has a tendency to be overly optimistic. The apparent simplicity may conceal the fact that important assumptions are being made when undertaking the bootstrap analysis (e.g. independence of samples) where these would be more formally stated in other approaches. The number of bootstrap samples recommended in the literature has increased as available computing power has increased. If the results really matter, as many samples as is reasonable given available computing power and time should be used. Increasing the number of samples cannot increase the amount of information in the original data, it can only reduce the effects of random sampling errors which can arise from a bootstrap procedure itself. We did not bootstrap our samples and the training and validation neural networks were computed based on randomly assigned samples performed just once. See on Cross-validation the justification.

**Odds and Odds Ratio:** the odds of an event is the probability of its occurrence divided by the probability that it will not occur. The ratio of one odds to another gives the odds ratio. When logistic regression is used to compute predictors for events, the odds ratio for a given predictor is the ratio of the event odds in the presence of the predictor divided by the event odds in its absence. Exponentiation of the regression coefficient of the predictor variable to the base *e*, enables the odds ratio being obtained. In the Medical Literature, readers are familiar with odds ratios for significant predictors variables along their 95% confidence intervals. An extensive comparison of standard logistic versus neural network models in short-term predictors of Type A AAD has been previously performed [2]. It is important to point out that with neural networks it is not possible to provide individual odds for the predictors, due to the non probabilistic nature of the assessment in the relation between potential predictors and binary events.
