# Supplementary material for: Long-term mortality prediction after operations for type A ascending aortic dissection
Source: J Cardiothorac Surg. 2010 May 25;5:42. doi: 10.1186/1749-8090-5-42 (PMC2902218; doi:10.1186/1749-8090-5-42)
Supplement: Additional file 2 — Table S1: Description and univariate contribution of 32 potential risk factors. [file 1749-8090-5-42-S2.DOC]

**Table S1. Description and univariate contribution of 32 potential risk factors.**

____________________________________________________________________________________________________________________________

**ID Code Variable (value) Min Max Mean±SD % Miss** **Information Value Gini** **Linear Correlation**

____________________________________________________________________________________________________________________________

**Demographic**

2 Annint Year of surgery 2002 2008 2005±2 0.0 0.012 0.007 0.008

23 HOSP Hospital (0=Rome; 1=Catanzaro) 0 1 0.39±0.48 0.0 0.022 0.072 0.071

1 Age Age (years) 27 85 62.91±11.76 0.0 0,654 0.272 0.244

30 Sex Sex (0=female; 1=male) 0 1 0.63±0.48 0.0 0.001 0.014 0.014

**Pre-operative**

25 PoIp High blood pressure (0=no; 1=yes) 0 1 0.85±0.36 0.0 0.033 0.029 0.036

26 PoMD Marfan syndrome (0=no; 1=yes) 0 1 0.06±0.24 0.0 0.073 0.062 0.122

27 PoSk Shock (0=no; 1=yes) 0 1 0.21±0.41 0.0 0.200 0.188 0.220

28 PoTOT Intubation (0=no; 1=yes) 0 1 0.10±0.30 0.0 0.102 0.100 0.159

24 PoDN Neurological symptoms (0=no; 1=yes) 0 1 0.08±0,28 0.0 0.001 0.009 0.015

29 Redo Previous cardiac surgery (0=no; 1=yes) 0 1 0,04±0.20 0.0 0.090 0.063 0.151

**Operative**

31 TCEC Extracorporeal circulation (min) 24 682 213.06±75.97 1.3 0.713 0.355 0.309

3 Arrc Circulatory arrest (min) 0 200 41.80±32.91 1.3 0.684 0.306 0.285

32 VAB Bicuspid aortic valve (0=no; 1=yes) 0 1 0.02±0.14 0.0 0.001 0.004 0.013

11 HP Hemopericardium (0=no; 1=yes) 0 1 0,42±0.49 0.0 0.002 0.023 0.023

**Immediate post-operative**

4 Bl24 Bleeding in the first post-operative 24 hours (ml) 0 6710 987.22±828.24 9.4 0.790 0.103 0.106

19 IscL Limb ischemia (0=no; 1=yes) 0 1 0.10±0.30 8.9 0.033 0.057 0.085

6 Cren Renal complications (0=no; 1=yes) 0 1 0.26±0.44 8.9 0.487 0.320 0.330

7 Cvvh Dialysis in continuous (0=no; 1=yes) 0 1 0,21±0.40 8.9 0.524 0.309 0.346

18 IRC Chronic renal failure (0=no; 1=yes) 0 1 0,07±0.26 8.9 0.803 0.231 0.401

5 Cint Enterological complications (0=no; 1=yes) 0 1 0,04±0.21 8.9 0.042 0.046 0.100

**Dummies for operative techniques and related complications**

12 dInt1 Dummy for surgery on ascending aorta (0=no; 1=yes) 0 1 0,42±0.49 0.0 0.012 0.054 0.052

13 dInt2 Dummy for surgery on ascending aorta plus hemiarch (0=no; 1=yes) 0 1 0.12±0.33 0.0 0.113 0.106 0.152

14 dInt3 Dummy for surgery on ascending aorta plus arch (0=no; 1=yes) 0 1 0.10±0.30 0.0 0.021 0.045 0.071

15 dInt4 Dummy for surgery on ascending aorta plus aortic valve (0=no; 1=yes) 0 1 0.17±0.30 0.0 0.006 0.031 0.039

16 dInt5 Dummy for surgery according to Bentall (0=no; 1=yes) 0 1 0.14±0.35 0.0 0.001 0.009 0.012

17 dInt6 Dummy for surgery according to Cabrol (0=no; 1=yes) 0 1 0.01±0.12 0.0 0.042 0.029 0.108

20 dPerBr1 Dummy for operative retrograde brain perfusion (0=no; 1=yes) 0 1 0.41±0.49 0.4 0.037 0.094 0.092

21 dPerBr2 Dummy for operative anterograde brain perfusion (0=no; 1=yes) 0 1 0.55±0.49 0.4 0.009 0.047 0.046

22 dPerBr3 Dummy for operative antero-retrograde brain perfusions (0=no; 1=yes) 0 1 0.02±0.17 0.4 0.067 0.047 0.132

8 dDN0 Dummy for no post-operative neurological deficit (0=no; 1=yes) 0 1 0.82±0.37 8.9 0.546 0.295 0.355

9 dDN1 Dummy for post-operative focal neurological deficit (0=no; 1=yes) 0 1 0.11±0.31 8.9 0.111 0.111 0.160

10 dDN2 Dummy for post-operative neurological coma (0=no; 1=yes) 0 1 0.06±0.23 8.9 0.587 0.184 0.351

**Out Status Alive (=0) or dead (=1) at long-term** 0 1 0.36±0.48 0.0

____________________________________________________________________________________________________________________________

There were 235 patients (24 with input data missing) operated for Type A acute aortic dissection, between 2002 and 2008, at the University of Rome “La Sapienza” and Catanzaro Sant’Anna Hospital, Cardiac Surgical Departments: 84 deaths (36%) were observed an average of 564±48 (mean±SE) days after operation.

SE=standard error; SD=standard deviation. Linear correlation is against Status. With dichotomous variables n is obtained by multiplying 235 by mean.
