# Supplementary material for: Long-term mortality prediction after operations for type A ascending aortic dissection
Source: J Cardiothorac Surg. 2010 May 25;5:42. doi: 10.1186/1749-8090-5-42 (PMC2902218; doi:10.1186/1749-8090-5-42)
Supplement: Additional file 3 — Model2Chronic.html. This is a tool to use the multivariable assessment of long-term mortality in Type A AAD patient, as obtained in this investigation. [file 1749-8090-5-42-S3.HTM]

By philbrierley.com


This is a Neural Network based prediction of long-term outcome in Type A aortic dissection patients.  


|  |  |  |  |
| --- | --- | --- | --- |
|  |  |  |  |
|  |  | Min Exp | Max Exp |
| Age |  | 27 | 85 |
| Annint |  | 2002 | 2008 |
| Arrc |  | 0 | 200 |
| Bl24 |  | 0 | 6710 |
| Cint |  | 0 | 1 |
| Cren |  | 0 | 1 |
| Cvvh |  | 0 | 1 |
| dDN0 |  | 0 | 1 |
| dDN1 |  | 0 | 1 |
| dDN2 |  | 0 | 1 |
| HP |  | 0 | 1 |
| dInt1 |  | 0 | 1 |
| dInt2 |  | 0 | 1 |
| dInt3 |  | 0 | 1 |
| dInt4 |  | 0 | 1 |
| dInt5 |  | 0 | 1 |
| dInt6 |  | 0 | 1 |
| IRC |  | 0 | 1 |
| IscL |  | 0 | 1 |
| dPerBr1 |  | 0 | 1 |
| dPerBr2 |  | 0 | 1 |
| dPerBr3 |  | 0 | 1 |
| HOSP |  | 0 | 1 |
| PoDN |  | 0 | 1 |
| PoIp |  | 0 | 2 |
| PoMD |  | 0 | 1 |
| PoSk |  | 0 | 1 |
| PoTOT |  | 0 | 1 |
| Redo |  | 0 | 1 |
| Sex |  | 0 | 1 |
| TCEC |  | 24 | 682 |
| VAB |  | 0 | 1 |
|  |  |  |  |
| Prediction |  || Status |  | Strength = |  |
|  |  |  |  |

**'Max/Min Exp'** - the maximum and minimum values experienced during the creation of the model. Visual warnings are given if your input values are outside this range of experience.
  
**'Strength'** - this is based on a scale from -1 to +1, where 0 is a borderline case and +1 is the best TRUE case and -1 is the best FALSE case on the training data. The higher the absolute value of the number, the stronger the classification.


---

|  |  |
| --- | --- |
| inputs: | 32 |
| hidden neurons: | 2 |
| training data source: | G:\DATI-PENDR\Mac1\DIS2.xls |
| training data view: | Foglio1$ |
| Tiberius weights file: | G:\PAPERSandSCIENCE\Papers0910\EurJCThSurg\Model2Chronic.twf |
| model created: | ven, nov 27 2009, 2.59 |
| this page created: | lun, gen 11 2010, 11.42 |

---

Visit the Tiberius Web Site
